# Resilience, trust, and civic engagement in the post-CCSVI era

**DOI:** 10.1186/s12913-018-3130-x

**Published:** 2018-05-16

**Authors:** Shelly Benjaminy, Andrew Schepmyer, Judy Illes, Anthony Traboulsee

**Affiliations:** 1 Neuroethics Canada, University of British Columbia, Vancouver, Canada; 2 Division of Neurology, Department of Medicine, University of British Columbia, Vancouver, Illinois Canada; 3 Current: Shirley Ryan AbilityLab, Chicago, Illinois USA

**Keywords:** Health services research, Public engagement, Multiple sclerosis, Chronic cerebrospinal venous insufficiency, Ethics

## Abstract

**Background:**

Scientific and financial investments in chronic cerebrospinal venous insufficiency (CCSVI) research have been made to address both the hope for and scepticism over this interventional strategy for MS. Despite limited evidence in support of the CCSVI hypothesis, the funding of clinical research was responsive to a demand by the public rarely seen in the history of medicine. We characterize patient perspectives about the CCSVI research trajectory, with particular attention to its impact on other non-pharmaceutical areas of MS research with a focus on stem cell interventions.

**Methods:**

Semi-structured interviews with 20 MS patients across Canada who did not have CCSVI interventions. Interviews were analysed for recurring themes and individual variations using the constant comparative approach.

**Results:**

Participants had a critical view of the divestment of funds from longstanding research to support CCSVI trials. They retain a sense of optimism, however, about emerging evidence for stem cell interventions for MS, and highlight the need for greater caution and conscientious communication of advances in medicine and science.

**Conclusions:**

The unrealized hopes for CCSVI challenged but did not undermine the resilience of patient communities. The narrative that unfolded highlights the importance of drawing a socially-minded space for public participation in science.

## Background

The chronic cerebrospinal venous insufficiency (CCSVI) hypothesis became a focus for multiple sclerosis (MS) research when a small study suggested that an angioplasty-like procedure would restore efficient haemodynamic flow, reduce iron deposits in the brain, and prevent related inflammation and myelin sheath attack [1, 2]. The hope for CCSVI was particularly pronounced in North America, where the MS community embarked on an unprecedented venture to secure investment in related research in hopes of a cure. Despite initial studies that challenged the CCSVI hypothesis [3, 4], the intervention—sometimes termed *liberation therapy*—was prevalent in the public sphere through wide-reaching anecdotal accounts of therapeutic gain described in social media, and through advocacy efforts that pressured policy makers to mobilize access to the public [5].

In the current era of biomedicine in which models of public participation in science are embraced and a landscape of increasingly accessible social media is democratizing science, the voices of the public were heard. Indeed, despite widespread caution from experts, over $20 million were invested in CCSVI research in Canada and the United States [5]. Severe adverse events and preventable fatalities from the CCSVI procedure were reported by others [6, 7]. Recent results from a multi-site Canadian CCSVI clinical trial demonstrate negative results in 104 MS patients [8].

In an environment in which health research resources are taxed [9], investment in CCSVI research diverted both funding and attention from other areas of clinical inquiry. Here we examine the lessons learned from the CCSVI research experience to understand the impact of the deviation in research on the perspectives of patients with MS and their trust in biotechnology. We focus on stem cell research in particular, an area of non-pharmaceutical inquiry that has been at the heart of hope in the MS community for nearly 30 years, and that has produced promising contemporary results through both hematopoietic and mesenchymal approaches [10–13].

## Methods

We recruited individuals with MS from across Canada, a country in which MS prevalence – with nearly 100,000 affected individuals – exceeds others internationally, and where advocacy efforts for CCSVI research were particularly widespread and influential [5]. We used a convenience sampling approach where research participants were recruited through online advertisements on patient advocacy group websites and in person through MS clinics. Participation refusal rates are not reported here, due to the nature of self-selection in the study design. None of the enrolled participants dropped out of the study. The time interval for participation was between May 2014 and August 2016. Inclusion criteria were: a diagnosis of MS; age ≥ 19 years; ability to provide informed consent; and, ability to speak English.

Following approval by the University of British Columbia Research Ethics Board (H13–03275) and standard procedures for acquiring informed written consent, we conducted a series of in-depth semi-structured interviews. The interview guide was informed by past studies of patient perspectives about novel biotechnologies [14, 15]. The overarching research question was: What are the perspectives of MS patients about the CCSVI research trajectory, and how do these impact their views on stem cell research?

The interview guide was initially designed to focus on about stem cell research, but, as prominent themes about the impact of CCSVI arose, we adapted the interviews to incorporate content that was responsive to participant priorities. This flexibility is consistent with the inductive approach described by Charmaz [16] that accommodates and respects the emic tradition of qualitative inquiry. We probed for familiarity with the CCSVI research trajectory, perspectives about CCSVI research and its impact, and perspectives about the promise of stem cell interventions as a potential therapeutic target for MS. One investigator (S.B.) conducted all interviews over the phone or in person and took detailed field notes. S.B. is a female PhD student with graduate level training in bioethics and qualitative inquiry. She had no pre-existing relationships with research participants prior to study commencement. Research participants were informed about S.B.’s position at the University of British Columbia, and the objectives of the study were explained in the consent process prior to interview commencement. Interviews were audio-recorded for transcription. We interviewed participants until no new major themes were identified from additional interviews and theoretical saturation was reached [16]. Verbatim transcripts of interviews were made software-ready and managed using NVivo 11 qualitative analysis software. Analysis was conducted in conjunction with ongoing data collection.

Using standard qualitative inquiry methods and iterative and deliberative approach, S.B. and A.S. developed a codebook that reflected the emerging phenomena and the hierarchy of themes and subthemes in the data set. Data were analyzed line by line for initial codes, which were then organized into major themes and subthemes through a constant comparative approach to characterize recurring phenomena and individual variations within and between transcripts [16]. To ensure dependability of the primary codes, 10% of the sample was coded independently by S.B. and A.S. A Cohen’s kappa test was performed on this sample size using the coding comparison feature in NVivo 11. The coding comparison feature in NVivo 11 is designed to measure inter-coder agreement in qualitative data while accounting for agreement by chance. This analysis yielded a Cohen’s kappa coefficient of 0.92, indicating substantial inter-coder agreement [17]. To ensure that the analysis of data represents the views of research participants, we provided all participants with synthesized study results representing major themes and illustrative quotes. We invited participants to comment on the data analysis and provide feedback on our interpretation of the data. Three participants responded to this call and reported back that, in their opinion, the data analysis captured perspectives authentically. Respondents’ suggestions to ensure diversity among illustrative quotes were integrated into the final analysis.

We interviewed 20 individuals who have MS (Table 1). None of the participants received a CCSVI intervention. Interviews ranged between 23 min and 80 min in length, for a total of 13.9 h of audio-recorded data for analysis. We focus here on the four major themes – grasping on to hope, costs of CCSVI research, enduring optimism, lessons learned – and eight subthemes (Table 2) that were generated by participant narratives. Major themes were defined by their prominence and relevance to the objectives of the study.

**Table 1:** Participant characteristics (*n* = 20)

Characteristic	Number of participants (%)
Gender	
Male	7 (35)
Female	13 (65)
Age (years)	
19–30	6 (30)
31–40	4 (20)
41–50	5 (25)
51–60	3 (15)
61–70	2 (10)
Education	
Grade school	1 (5)
High school	4 (20)
College	8 (40)
University	4 (20)
Advanced degree (e.g., MD, PhD, JD)	3 (15)
MS sub-type	
Relapsing remitting	10 (50)
Primary progressive	1 (5)
Secondary progressive	7 (35)
Other variant^b^	2 (10)
Time since MS diagnosis (years)	
0–5	5 (25)
6–10	5 (25)
11–15	6 (30)
16–20	3 (15)
21+	1 (5)
Sources of information about MS^a^	
Neurologist	20 (100)
Family physician	7 (35)
Other clinician	7 (35)
Internet forums	10 (50)
Internet health sites	12 (60)
Newspapers	5 (25)
Magazines	5 (25)
Television	6 (30)
Support groups	13 (65)
Other	10 (50)

^a^Non-mutually exclusive categories

^b^Rare variants of MS including Marburg MS

**Table 2:** Emergent themes

Themes and sub-themes	Description
Grasping onto hope Initial hopes Disappointment	Initial hopes and subsequent disappointment about chronic cerebrospinal venous insufficiency research in the multiple sclerosis community
Costs of chronic cerebrospinal venous insufficiency research Medical adverse events Divestment of research funds Limited efficacy Translational delays	Adverse implications of chronic cerebrospinal venous insufficiency research
Enduring optimism Caution in the wake of disappointment Forging onward with stem cell research	Retained optimism and continued support for stem cell research to address multiple sclerosis
Lessons learned	Knowledge gleaned from the chronic cerebrospinal venous insufficiency experience that may shape future scientific endeavours

## Results

### Grasping onto hope

Participants described the sense of hope and desperation in the community at the time that the CCSVI hypothesis was proposed. They articulated how hope motivated some MS patients to seek access to CCSVI interventions prior to sufficient testing, many times through clinics that offer unregulated interventions abroad, and often against medical advice.

*There were a lot of people grasping on to hope... Desperation definitely played a part in it…A lot of people [were] willing to have the procedure [CCSVI] done prior to having North American testing done.*

—Participant 8 (Female, relapsing remitting MS)

### Costs of CCSVI research

Participants described the disappointment that was felt in the MS community following premature access to the procedures, and the costs of CCSVI research. Many explained that the intervention did not yield lasting medical benefits, and some described the procedure as a temporary fix. Participants explained that those who experienced a temporary sense of wellness after CCSVI procedures felt the most disappointment.

*I think people go for [CCSVI] and probably the most disappointment [is felt by] the people who have it and six months later they're right back to where they were.*

—Participant 17 (Female, secondary progressive MS)

Participants described the adverse events endured by some patients who received unregulated CCSVI interventions abroad, and were also critical about the financial investment in CCSVI research. These participants explained that money that could have been invested in scientifically bolstered MS interventions, such as stem cell research, was spent on CCSVI research. Few participants were particularly critical about this divestment of funds, citing knowledge of scientific evidence that brought the CCSVI hypothesis into question prior to the funding of clinical trials.

*It's just wasted money, especially when it's a disproved theory, when that money could have gone to better use to support research for the stem cell area*.

—Participant 20 (Male, other MS variant)

Participants explained that the time spent on CCSVI research would have been better spent on other areas of inquiry, such as stem cell research. In addition, they worried that the lack of credibility associated with CCSVI research would infiltrate the public domain and undermine confidence among the MS community in the scientific enterprise. Participants suggested that this would necessitate more rigorous testing in the stem cell arena, and could result in delayed translation.

*It makes me sad that it [CCSVI research] turned into such a fiasco cause…it’ll [stem cell research] take longer and it’ll take more proving…We wouldn’t have to work so hard to prove [stem cell research] if we hadn’t have shot ourselves in the foot with CCSVI first.*

—Participant 14 (Female, other MS variant)

### Enduring optimism

Despite disappointment with CCSVI research, all participants still articulated optimism that stem cell research would yield a treatment for MS. However, optimism was guarded and cautious.

*Five years ago it [CCSVI] came out and a lot of people saw it as a cure, and so everybody jumped on that bandwagon…So I didn’t want to put all my cards on that table. And I’m at sort of that point with stem cell. I’m hopeful and eager to see what happens, but I’m not ready to jump into it.*

—Participant 5 (Female, relapsing remitting MS)

Participants explained that optimism for stem cell research, unlike those that supported CCSVI research, are based in trusted science. They explained that this is why they continue to support stem cell research.

*I think CCSVI was anecdotal…[stem cell research] has hard science behind it…they’ve been researching it for many years in relation to different uses.*

—Participant 1 (Female, relapsing remitting MS)

Participants explained that they continue to support the development of research that tackles MS. Some expressed uncertainty associated with medical research with the view that there are no guarantees in the pursuit of knowledge, and that scientists must move forward despite setbacks to find new treatment options.

*It was a disappointment but…I knew it wasn’t a guarantee…if it works it works and if it doesn’t, well, let’s go forward.*

—Participant 6 (Male, secondary progressive MS)

### Lessons learned

Participants explained that society must reflect on the CCSVI experience to learn lessons about how to prioritize research in the future. They suggested that scientists ensure that research is safe before the public accesses it.

*I would say that before you started treatment on the patient…you should be…sure that it’s very safe to try it.*

—Participant 12 (Male, secondary progressive MS)

They also suggested that scientists must promote social responsibility in science communications. They underscored the importance of public trust for the sustainability of the research enterprise, pointing to the joint responsibility of both the news media and scientists.

*The media needs to be more responsible to what they present openly…That [CCSVI] was pretty devastating for people.*

—Participant 11 (Female, secondary progressive MS)

*…[scientists should] not to jump the gun and say…“we found a new cure for MS…come and try this”, and then it doesn't work…because…people get their hopes up, and then…it's just going to damage the [community’s] view of [research].*

—Participant 20 (Male, other MS variant)

## Discussion

The CCSVI story represents a historical moment in biomedicine and health care research that highlights the challenges of prioritizing a responsive and conscientious space for patient advocacy and public participation in science and policy. The MS community was faced with a profound dilemma: how to integrate genuine hope in the public sphere into science policy and funding priorities while also accounting for the absence of evidence-based data [5, 18]. While the force of the CCSVI movement was exceptional, reminiscent perhaps only of few others such as the HIV movement in the 1980s, the effect public endorsements for access to biomedical research have been a more frequent topic of discussion. They have implicated a wide range of applications, such as genetics and genomics research [14, 19], personalized medicine [20], stem cell research [21, 22], and neuroimaging [23, 24]. This literature repeatedly predicts the theoretical outcomes of sensationalism: it may leave stakeholders hopeful and vulnerable to undue disappointment and distress [25], and cycles of inflated expectations and subsequent disappointment may create unsustainable links in the chain of translation for novel biomedical research, disillusionment among clinicians, despair among patient communities, loss of public trust in science, and the disengagement of industry [20, 26, 27].

Few studies, however, have examined empirically the implications of these messages on patient communities who have great stakes in the investment and outcomes of the research [28]. Even in the face of negative trials [6], the findings here are less disheartening: they point to community resilience and enduring optimism for research, including novel stem cell interventions. In contrast with MS patient narratives that unveil a lack of trust among those who chose to pursue unproven and unregulated stem cell interventions outside of their home countries for what is often referred to as medical tourism [29], participants in this study retained their trust in science and urged the medical community to continue research efforts in the hopes of finding effective interventions for MS.

Contemporary formulations of science are increasingly moving towards more pluralistic approaches that encourage public participation in science. These include research methods that celebrate porous and reciprocal engagement between scientists and the public such as deliberative democracies and participatory action research, as well as government funding priorities that include knowledge translation, mobilization, and exchange initiatives that encourage engagement between scientists and lay citizens [30]. The imperative of democratizing science is not only logical, but also socially conscientious, as the citizens who bear the costs and burdens of scientific advance should be informed and involved in its development and application [31]. Moreover, public engagement in science and science policy aligns with the values of accountability and transparency, and is thought to be a tool for promoting public trust in technology development [32, 33].

This study demonstrates that public engagement in science, while an ethical imperative, is not without its challenges. In the CCSVI context, patient advocacy unveiled divergence between the scientific community that valued evidence-based medicine, and patient priorities for timely access to potentially life saving interventions in the face of scientific uncertainty. Participant-drawn lessons reinforce the imperative for conscientious communication of advances in medicine and science and mutual responsibilities. While participants advocated for social responsibility – in fact, a duty for top-down communication [23, 34, 35] – they reflected considerably less about the MS community’s roles and responsibilities in influencing views and policy. Yet, CCSVI research gained significant momentum by way of anecdotal accounts of patients who underwent the procedure through the social media platforms such as YouTube and the blogosphere [18, 36]. Such public-generated endorsements led to significant political pressure that shaped science policy and mobilized funding for CCSVI clinical trials [5]. Indeed, the CCSVI story teaches that patients are eager to engage, increase knowledge about their disease, and exchange advice with other patients [37], but that much work remains to be done in closing a gap that still exists in supporting them well to do so.

## Limitations

As is standard in the tradition of qualitative inquiry, this study has limitations. It cannot aim to be generalizable. Rather it yields transferable data that illustrate the perspectives of a sample of MS patients during the time at which this study was conducted. Clear trends in participant perspectives did not segregate by demographic criteria (e.g., MS subtype, gender, or education). Such trends may be masked by the limited sample size in this study or by the self-selection element in the recruitment strategy. Moreover, the perspectives presented in this study only represent views of patients who did not receive CCSVI interventions. The data were collected over a two-year timeframe between 2014 and 2016 – an interval during which clinical trials for the CCSVI procedures were ongoing, but results still unknown. An exploration of perspectives after the results of the trial are published will reveal further important considerations for science policy.

## Conclusion

Overall, MS patient community is resilient, hopeful, and trusting of ongoing developments in biotechnology. Participant perspectives draw attention to lingering challenges in translation of biomedical research from the bench to the bedside, including the imperative for carefully balancing civic engagement and scientific evidence. Indeed, the CCSVI research trajectory demonstrates that democratizing science is not without risks and challenges. The CCSVI experience serves as an opportunity for reflection, and enriches the field of biomedicine with invaluable lessons about the complex relationship between science and society.
